# Somatic survival and organ donation among brain-dead patients in the state of Qatar

**DOI:** 10.1186/s12883-016-0719-8

**Published:** 2016-10-31

**Authors:** Saibu George, Merlin Thomas, Wanis H. Ibrahim, Ahmed Abdussalam, Prem Chandra, Husain Shabbir Ali, Tasleem Raza

**Affiliations:** 1 Medical Intensive Care Department, Hamad Medical Corporation, Doha, Qatar; 2 Pulmonary Department, Hamad Medical Corporation, Doha, Qatar; 3 Medical Research Centre, Hamad Medical Corporation , Doha, Qatar; 4 Internal Medicine, Hamad Medical Corporation, Doha, Qatar

**Keywords:** Brain dead, Somatic survival, Organ donation

## Abstract

**Background:**

The Qatari law, as in many other countries, uses brain death as the main criteria for organ donation and cessation of medical support. By contrast, most of the public in Qatar do not agree with the limitation or withdrawal of medical care until the time of cardiac death. The current study aims to examine the duration of somatic survival after brain death, organ donation rate in brain-dead patients as well as review the underlying etiologies and level of support provided in the state of Qatar.

**Methods:**

This is a retrospective study of all patients diagnosed with brain death over a 10-year period conducted at the largest tertiary center in Qatar (Hamad General Hospital).

**Results:**

Among the 53 patients who were diagnosed with brain death during the study period, the median and mean somatic survivals of brain-dead patients in the current study were 3 and 4.5 days respectively. The most common etiology was intracranial hemorrhage (45.3 %) followed by ischemic stroke (17 %). Ischemic stroke patients had a median survival of 11 days. Organ donation was accepted by only two families (6.6 %) of the 30 brain dead patients deemed suitable for organ donation.

**Conclusion:**

The average somatic survival of brain-dead patients is less than one week irrespective of supportive measures provided. Organ donation rate was extremely low among brain-dead patients in Qatar. Improved public education may lead to significant improvement in resource utilization as well as organ transplant donors and should be a major target area of future health care policies.

## Background

Permanent cessation of all brain functions that entails cessation of cerebral and brain stem functions clinically define the term “whole brain death” [1]. Regardless of the persisting function of other individual organs, whole brain death that results in permanent loss of brain function, loss of consciousness, cognition and respiratory drive is the hallmark of irreversible cessation of the function of the organism as a whole [2]. The survival of these patients post confirmation of brain death is termed as “somatic survival” and this is usually maintained with the help of breathing and or circulatory support. Despite the wide-spread acceptance of brain death as death on clinical, ethical and legal grounds, the concept remains vague to many physicians as well as to the public [2]. A number of publications have questioned the use of brain death as clinical and legal death on ethical grounds [3–6]. Qatar is a small country with Islam being the dominating religion. According to the Qatari law, brain death is used as criteria for organ donation and cessation of medical support. However, most of the public in Qatar do not agree to the limitation or withdrawal of medical care until the time of cardiac death. The current study aims to examine the duration of somatic survival after brain death, organ donation rate in brain-dead patients as well as review the underlying etiologies and level of support provided to these patients.

## Methods

This is a retrospective study of brain-dead individuals conducted at the largest governmental tertiary center in the state of Qatar (Hamad General Hospital) over a ten year period (January 2003 to December 2013). Eligible patients for the study were individuals aged ≥18 years with confirmed brain death (defined by Hamad General Hospital Brain Death Criteria that was largely adopted from the American Academy of Neurology (AAN) criteria [7]) who were admitted to Hamad General Hospital during the study period. Both electronic and non-electronic medical records were searched for parameters such as demographic characteristics, the length of somatic survival after brain death, the etiology of brain death, results of family consenting for organ donation and the rate of organ donation. Data were collected using an approved form. All Statistical analyses were done using statistical packages SPSS 22.0 (SPSS Inc. Chicago, IL). Qualitative and quantitative data values were expressed as frequency along with percentage and mean ± standard deviation with median and range. Descriptive statistics were used to summarize demographic, laboratory and all other clinical characteristics of the patients. Associations between two or more qualitative or categorical variables were assessed using chi-square test or Fisher exact test as appropriate. Quantitative variables means between two independent groups were analyzed using unpaired ‘*t*’ test or Mann Whitney U test as applicable. Univariate Kaplan–Meier survival analysis was applied to estimate median somatic survival in each group. Furthermore, the log-rank test was used to determine any statistical difference in median survival between the different groups. Pictorial presentations of the key results were made using appropriate statistical graphs. A two-sided *P* value <0.05 was considered to be statistically significant. The study was approved by the Medical Research Centre at Hamad Medical Corporation.

## Results

**Fig. 1:**
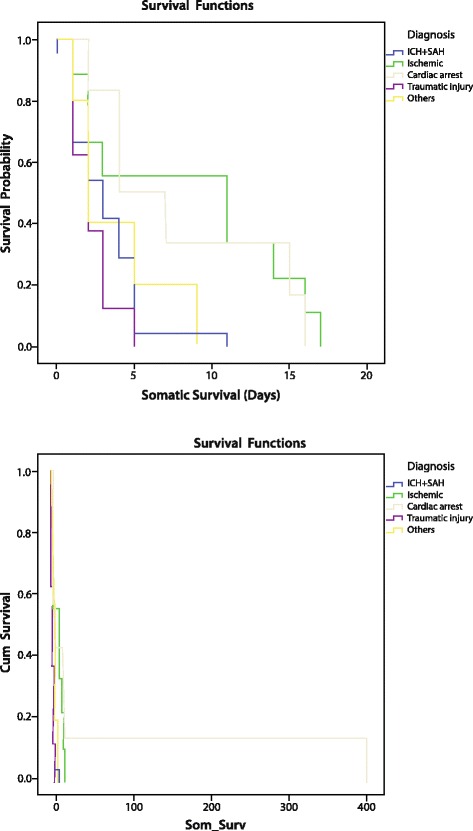
Somatic survival after brain death

Over the ten-year period of the study, 53 patients were diagnosed as brain-dead using Hamad General Hospital Brain Death Criteria. The characteristics of these patients are shown in Table 1. Majority (81.1 %) of patients were males. Thirty four (63 %) patients were admitted to medical Intensive Care Unit (ICU) and 11 (20.4 %) were admitted to Surgical ICU. The median and mean somatic survivals of brain-dead patients in the current study were 3 and 4.5 days respectively. Using the Kaplan-Meir survival analysis, brain-dead patients due to ischemic stroke, cardiac arrest (hypoxic encephalopathy), and intracranial hemorrhage had median somatic survivals of 11, 4, and 3 days respectively (Fig. 1). Confirmation of brain death was done within 24 h in 24 %, 48 h in 18 % and 3 days in 56 % of patients. The most common etiology for brain death was intracranial hemorrhage (45.3 %) followed by ischemic stroke (17 %) and traumatic brain injury (15.1) (Table 1). With regards to organ donation, only two families accepted organ donation. Thirteen cases were not appropriate donors in view of multiple comorbidities, cancer and infection. Family refused organ donation in 28 cases. In nine patients, declaration of patient death prior to assessment for organ donation was observed. In two cases, relatives could not be traced to obtain consent and one patient was a pregnant lady. Withdrawal of care was accepted by one family and for all other patients full intensive care inclusive of respiratory, hemodynamic, renal, nutritional, and nursing care support was provided and continued after discussion with patient’s families.

**Table 1:** Characteristics of patients with brain death

Characteristics	*n* (%)
Male	43 (81)
Female	10 (18.9)
Coronary care unit	1 (1.9)
Medical ICU	34 (63.0)
Trauma ICU	8 (14.8)
Surgical ICU	11 (20.4)
Organ donation	2 (6.6 %)
Etiology of brain death	
Intracranial haemorrhage (ICH) and Subarachnoid Haemorrhage (SAH)	24 (45.3)
Ischemic Stroke	9 (17)
Traumatic brain Injury	8 (15.1)
Cardiac arrest (Hypoxic encephalopathy)	5 (9.3)
Meningoencephalitis	2 (3.8)
Brain tumor with brain stem compression	1 (1.9)
Smoke inhalation with diffuse brain edema	1 (1.9)
Status asthmaticus	1 (1.9)
Brain astrocytoma	1 (1.9)

## Discussion

In 1995, the AAN published practice parameters for diagnosis of brain-death [7]. The parameters emphasize on irreversible coma (with a known cause), absence of brain-stem reflexes and irreversible apnea. The diagnosis of brain-death is a clinical one and supplementary tests are only recommended in the presence of confounding factors. The AAN issued an evidence-based guideline update in 2010 [8] that concluded absence of any published reports of recovery of neurologic function after a diagnosis of brain death using the 1995 AAN criteria. Following brain injury, the initial care is usually directed towards preservation and restoration of neuronal function to prevent more serious consequences such as brain death [9]. In Qatar, the strict criteria of the AAN are used for the diagnosis of brain death and require confirmation by two independent senior physicians. Organ donation and discontinuation of medical support are usually discussed with patient’s family after confirmation of brain death. The finding of a median somatic survival of 3 days in brain-dead patients in the current study is comparable with the findings in other countries. In a study of 609 brain-dead patients conducted in the United Kingdom, the median somatic survival of these patients was 3.5–4.5 days [10]. Another study conducted on 73 brain-dead patients in Taiwan found that 81 % of these patients developed cardiac asystole in 3 days and 97 % in 7 days despite continued cardiorespiratory support [11]. A recent study from Kuwait showed a median survival of 6 days in 40 brain dead patients. A meta-analysis of brain-dead patients who survived one week or longer found that the longest survivors were all young children. In addition, all patients aged more than 30 years survived for less than two and half months [12]. A study conducted by Wijdicks et al., revealed similar findings to the current study with regard to the timing of diagnosis of brain death (within 24 h of presumptive brain death in 30 % of the patients and within 3 days in 62 %) [13]. The most common etiology of brain death in the current study was primary structural brain damage, causes of which being intracranial hemorrhage (cerebral and subarachnoid), followed by ischemic stroke and traumatic brain injury. This finding with regard to the etiology is in consensus with findings from previous studies where direct traumatic injury to the head (e.g. road accident), subarachnoid hemorrhage and ischemic stroke were found to be the most common causes of brain death [14]. Other causes include intra-cerebral hemorrhage, hypoxic-ischemic encephalopathy and infections. These pathologies result in severe damage of the brain by causing cerebral edema and rise in intra-cranial pressure (ICP) that in turn reduce cerebral perfusion leading to trans-tentorial herniation and coning at the foramen magnum with damage to the brainstem as a consequence [15]. Many previously published case series found that traumatic brain injuries and intracranial hemorrhage to be the most common etiologies of brain death [13, 16]. Cardio-pulmonary arrest from other causes was responsible for 9 % of causes of brain death. Brainstem death is relatively rare in cardio-pulmonary arrest as the commonly affected parts of the brain are the cerebral cortex and cerebellum if resumption of circulation fails beyond 5 min [17, 18]. Being a center for organ retrieval and transplantation, organ donation rates among brain-dead patients in our hospital were low during the study period. Out of the total number of patients deemed suitable as organ donors, family refusal was observed in nearly 93 % of the cases. This is likely due to complex factors including religious, cultural, population dynamics (majority expatriates) and poor understanding of organ donation. This strongly points to the need to enhance public education with regards to organ retrieval and transplantation. Despite the fact that the concept of brain death was introduced more than 40 years ago and has been widely accepted, differences continue with its concept and justification [19]. A minority of health care individuals worldwide, still debate the importance of organ donation and mind the unintended consequences for dying patients such as diagnostic errors during expedited process of brain death prior to retrieval of transplantable organs [20]. In Qatar and other Gulf Cooperation Council (GCC), there is an increasing trend of end-stage liver and renal disease with high need for increased organ donors to fulfill the increasing demands [21, 22]. The acute need of rapid evaluation, family discussion and timely retrieval of organs in brain-dead patients cannot be overemphasized. While there is legal precedent for discontinuing life support over the family’s objection, many rightly advocate delay, education, support, and negotiation in such cases [23–26].

## Conclusion

The time of somatic survival after brain death in Qatar is relatively short and the rate of organ donation among brain-dead patients is low. Public education and guidance regarding brain death may lead to significant improvement in proper utilization of ICU resources. Furthermore, organ donation rates will likely improve with more public education about brain death and transplantation.

## Abbreviations

AAN: American Academy of Neurology
ICU: Intensive care unit
ICP: Intra-cranial pressure
GCC: Gulf Cooperation Council
